# Telmisartan Exerts Anti-Tumor Effects by Activating Peroxisome Proliferator-Activated Receptor-γ in Human Lung Adenocarcinoma A549 Cells

**DOI:** 10.3390/molecules19032862

**Published:** 2014-03-05

**Authors:** Juan Li, Lin Chen, Ping Yu, Bin Liu, Jiang Zhu, Ye Yang

**Affiliations:** 1 Thoracic Oncology Department, Sichuan Cancer Hospital, Chengdu, Sichuan 610041, China; 2 Oncology Department, Sichuan Provincial Hospital and Sichuan Academy of Medical Science, Chengdu, Sichuan 610072, China

**Keywords:** telmisartan, A549 cells, lung cancer, peroxisome proliferator-activated receptor-γ (PPARγ), intercellular adhesion molecule-1 (ICAM-1), matrix metalloprotease-9 (MMP-9)

## Abstract

Telmisartan, a member of the angiotensin II type 1 receptor blockers, is usually used for cardiovascular diseases. Recent studies have showed that telmisartan has the property of PPARγ activation. Meanwhile, PPARγ is essential for tumor proliferation, invasion and metastasis. In this work we explore whether telmisartan could exert anti-tumor effects through PPARγ activation in A549 cells. MTT and trypan blue exclusion assays were included to determine the survival rates and cell viabilities. RT-PCR and western blotting were used to analyze the expression of ICAM-1, MMP-9 and PPARγ. DNA binding activity of PPARγ was evaluated by EMSA. Our data showed that the survival rates and cell viabilities of A549 cells were all reduced by telmisartan in a time- and concentration-dependent manner. Meanwhile, our results also demonstrated that telmisartan dose-dependently inhibited the expression of ICAM-1 and MMP-9. Moreover, the cytotoxic and anti-proliferative effects, ICAM-1 and MMP-9 inhibitive properties of telmisartan were totally blunted by the PPARγ antagonist GW9662. Our findings also showed that the expression of PPARγ was up-regulated by telmisartan in a dose dependent manner. And, the EMSA results also figured out that DNA binding activity of PPARγ was dose-dependently increased by telmisartan. Additionally, our data also revealed that telmisartan-induced PPARγ activation was abrogated by GW9662. Taken together, our results indicated that telmisartan inhibited the expression of ICAM-1 and MMP-9 in A549 cells, very likely through the up-regulation of PPARγ synthesis.

## 1. Introduction

Peroxisome proliferator-activated receptor γ (PPARγ), belonging to the peroxisome proliferators-activated receptors (PPARs) family, is considered to be essential for modulating multiple physical and pathological processes, including lipid and glucose metabolism, inflammation and fibrosis [1,2,3,4,5,6,7]. Additionally, amount of studies also confirmed that PPARγ plays a critical in tumor proliferation and differentiation, apoptosis, invasion, angiogenesis and metastasis [8,9,10,11,12]. Specifically, PPARγ activation was effective in arresting the proliferation of dedifferentiated tumor cells [8,9,10,11,12]. 

Adhesion molecules are involved in intracellular signaling in several critical pathological processes, including tumor invasion, which is one of the most important features of malignant tumors [13,14,15,16,17,18]. Among adhesion molecules, intercellular adhesion molecule-1 (ICAM-1), a member of the immunoglobulin supergene family, is an inducible surface glycoprotein. The extracellular domain of ICAM-1 plays a key role in the transendothelial migration of leukocytes from the capillary bed into the tissue [18,19,20]. Some studies found that ICAM-1 may also promote the tumor cells breaking through the extracellular matrix (ECM) [21,22]. Meanwhile, the studies revealed that overexpression of ICAM-1 was associated with a greater risk of advanced lung cancers (stages III and IV) and more aggressive behaviors in other epithelial-derived cancers, including melanoma, gastric and breast [23]. A wealth of reports demonstrated that PPARγ contributed substantially to the regulation of ICAM-1 in variety of inflammatory conditions [24,25]. Furthermore, Arnold *et al.* showed that ICAM-1 expression was markedly downregulated by PPARγ activation in A549 cells [26]. Matrix metalloprotease-9 (MMP-9), one of member of the matrix metalloprotease family, is essential for the cancer cell metastasis and migration, which are the major characteristics of malignant tumors and the most important reasons causing death [27,28,29]. Amount of studies uncovered the role of PPARγ as a central player in the regulation of MMP-9 expression [30,31,32]. 

Telmisartan, a member of angiotensin II type 1 receptor blockers (ARBs), is usually used for the treatment of cardiovascular diseases, including hypertension and coronary artery disease (CAD) [33,34]. Recently, several studies indicated that telmisartan and irbesartan have PPARγ–activating properties and they have been considered to be the selective PPARγ modulators [35,36]. Taken together, the purpose of this study was to explore the anti-tumor value of telmisartan in lung cancer A549 cells, and the bio-mechanism involved.

## 2. Results and Discussion

### 2.1. Telmisartan Inhibits the Cell Survival Rates and Cell Viabilities of A549 Cells

**Figure 1:**
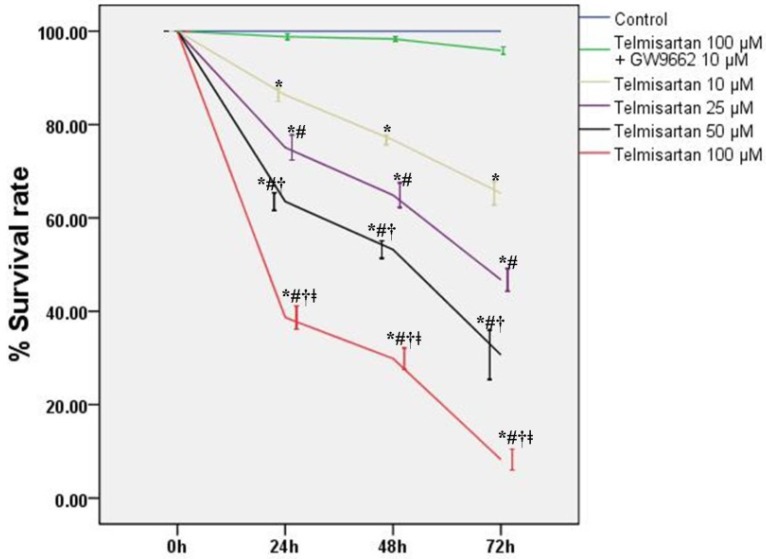
Telmisartan inhibits the survival rates of A549 cells. The survival rates of A549 cells was analyzed by MTT assay at specific time points (0, 24, 48, and 72 h) with 4 different concentrations of telmisartan (10, 25, 50 and 100 μM) or GW9662 (10 μM). Quantitative data are presented as mean ± SD (*n* = 4). * *p* < 0.05 compared with Control at the corresponding time points. # *p* < 0.05 compared with 10 μM telmisartan at the corresponding time points. † *p* < 0.05 compared with 25 μM telmisartan at the corresponding time points. ǂ *p* < 0.05 compared with 50 μM telmisartan at the corresponding time points.

**Figure 2:**
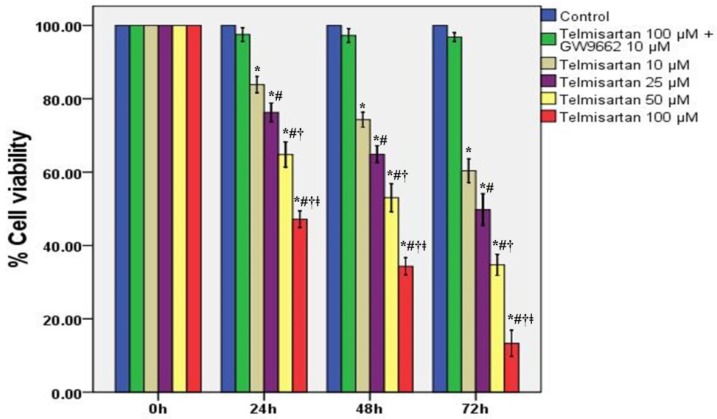
Telmisartan reduces the cell viability of A549 cells. The cell viability of A549 cells was analyzed by trypan blue exclusion assay at specific time points (0, 24, 48, and 72 h) with 4 different concentrations of telmisartan (10, 25, 50 and 100 μM) or GW9662 (10 μM). Quantitative data are presented as mean ± SD (*n* = 4). * *p* < 0.05 compared with Control at the corresponding time points. # *p* < 0.05 compared with 10 μM telmisartan at the corresponding time points. † *p* < 0.05 compared with 25 μM telmisartan at the corresponding time points. ǂ *p* < 0.05 compared with 50 μM telmisartan at the corresponding time points.

To detect the values of telmisartan on the cytotoxicity and proliferation of A549 cells, cells were treated by four different concentrations of telmisartan (10, 25, 50 and 100 μM) with or without GW9662 (10 μM). Our data indicated that both the survival rates and viabilities of A549 cells were reduced by telmisartan in a time- and concentration-dependent manner (Figure 1 and Figure 2). Additionally, Figure 1 and Figure 2 also demonstrated that GW9662 (10 μM) abrogated the cytotoxic and anti-proliferative effects of telmisartan (100 μM).

### 2.2. Telmisartan Dose-Dependently Reduces the mRNA and Protein Expression of ICAM-1 and MMP-9 in A549 Cells

**Figure 3:**
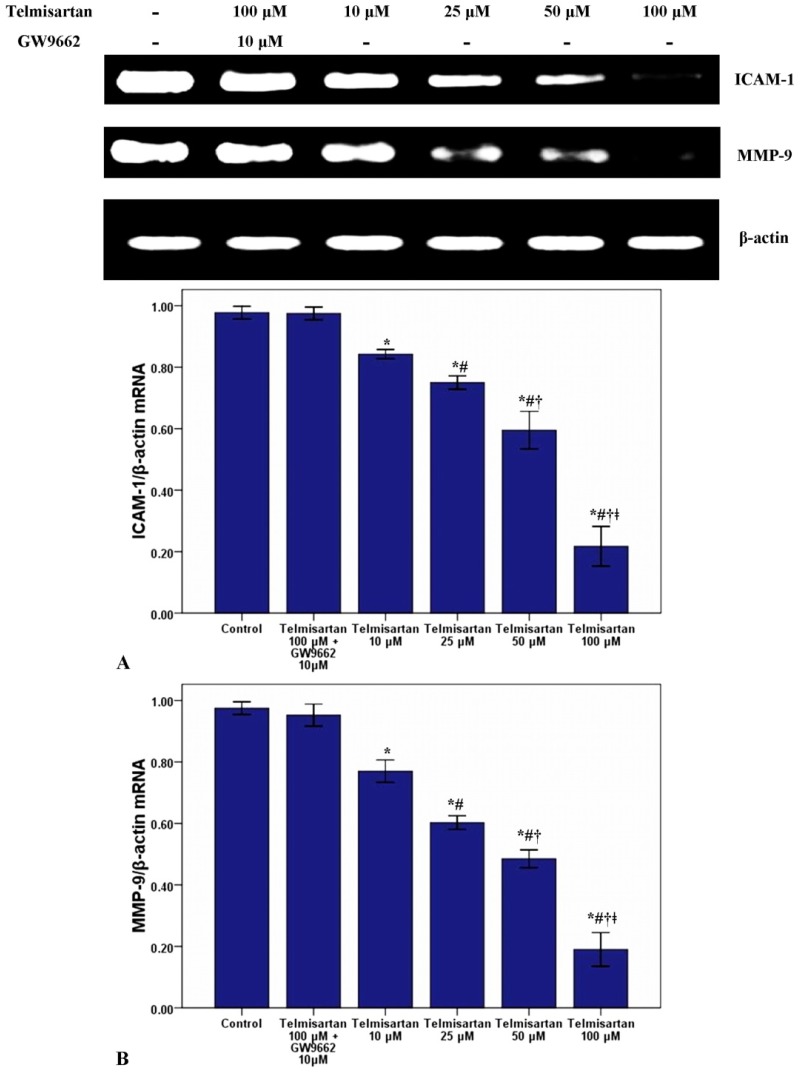
Telmisartan reduces the mRNA expression of ICAM-1 and MMP-9 in A549 cells. The mRNA expression of ICAM-1 (A) and MMP-9 (B) in A549 cells treated with 4 concentrations of telmisartan (10, 25, 50 and 100 μM) or GW9662 (10 μM) for 48 h were measured. Quantitative data were presented as mean ± SD (*n* = 4). * *p* < 0.05 compared with Control. # *p* < 0.05 compared with 10 μM telmisartan. † *p* < 0.05 compared with 25 μM telmisartan. ǂ *p* < 0.05 compared with 50 μM telmisartan.

**Figure 4:**
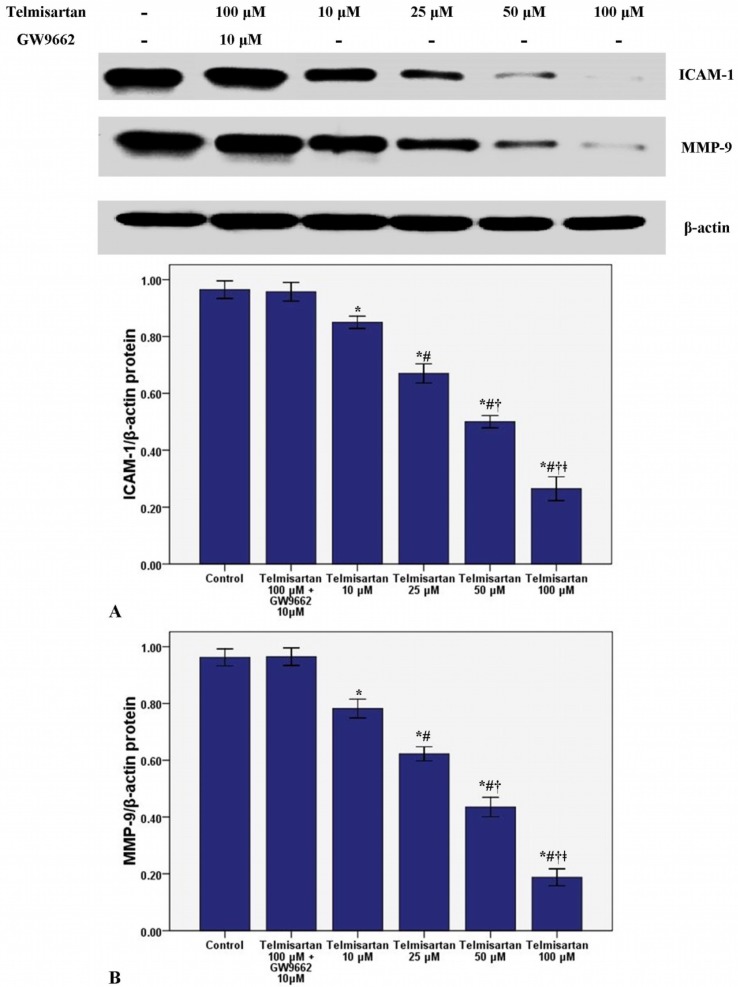
Telmisartan reduces the protein expression of ICAM-1 and MMP-9 in A549 cells. The protein expression of ICAM-1 (**A**) and MMP-9 (**B**) in A549 cells treated with 4 concentrations of telmisartan (10, 25, 50 and 100 μM) or GW9662 (10 μM) for 48 h were measured. Quantitative data were presented as mean ± SD (*n* = 4). * *p* < 0.05 compared with Control. # *p* < 0.05 compared with 10 μM telmisartan. † *p* < 0.05 compared with 25 μM telmisartan. ǂ *p* < 0.05 compared with 50 μM telmisartan.

To measure the effect of telmisartan on the mRNA and protein expression of ICAM-1 and MMP-9, RT-PCR and western blotting were used in our study. Figure 3 and Figure 4 showed that telmisartan dose-dependently inhibited the mRNA and protein expression of ICAM-1 and MMP-9. Meanwhile, our data also demonstrated that GW9662 blocked the effect of telmisartan on ICAM-1 and MMP-9 expression. 

### 2.3. Telmisartan Dose-Dependently Enhances the mRNA and Protein Expression of PPARγ in A549 Cells

**Figure 5:**
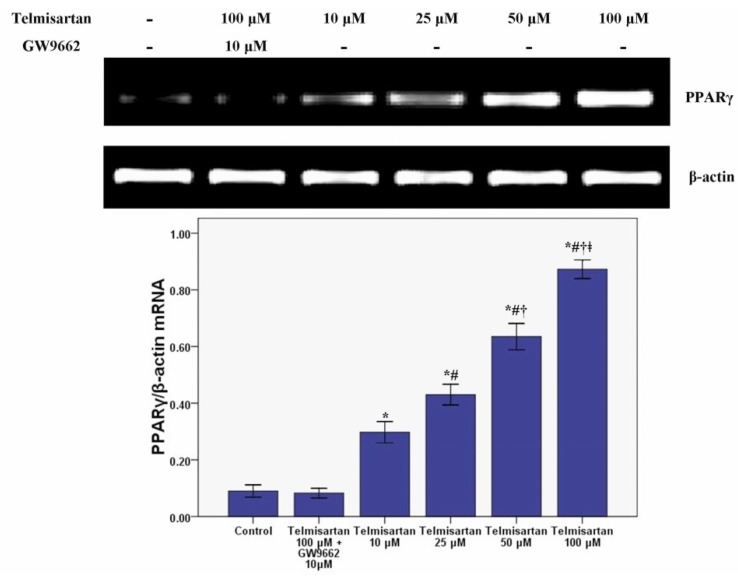
Telmisartan enhances the mRNA expression of PPARγ in A549 cells. The mRNA expression of PPARγ in A549 cells treated with 4 concentrations of telmisartan (10, 25, 50 and 100 μM)or GW9662 (10 μM) for 48 h were measured. Quantitative data were presented as mean ± SD (*n* = 4). * *p* < 0.05 compared with Control. # *p* < 0.05 compared with 10 μM telmisartan. † *p* < 0.05 compared with 25 μM telmisartan. ǂ *p* < 0.05 compared with 50 μM telmisartan.

**Figure 6:**
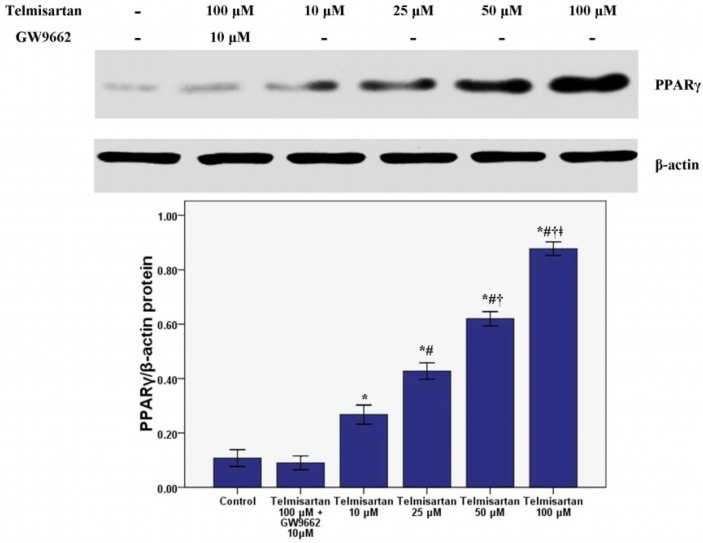
Telmisartan enhances the protein expression of PPARγ in A549 cells. The protein expression of PPARγ in A549 cells treated with 4 concentrations of telmisartan (10, 25, 50 and 100 μM) or GW9662 (10 μM) for 48 h were measured. Quantitative data were presented as mean ± SD (*n* = 4). * *p* < 0.05 compared with Control. # *p* < 0.05 compared with 10 μM telmisartan. † *p* < 0.05 compared with 25 μM telmisartan. ǂ *p* < 0.05 compared with 50 μM telmisartan.

RT-PCR and western blotting were performed to measure the mRNA and protein expression of PPARγ in our study. Our results revealed that the mRNA and protein expression of PPARγ was dose-dependently enhanced by telmisartan (Figure 5 and Figure 6). Meanwhile, our data also showed that GW9662 blocked the effect of telmisartan on PPARγ expression.

### 2.4. Telmisartan Increases DNA Binding Activity of PPARγ in A549 Cells

**Figure 7:**
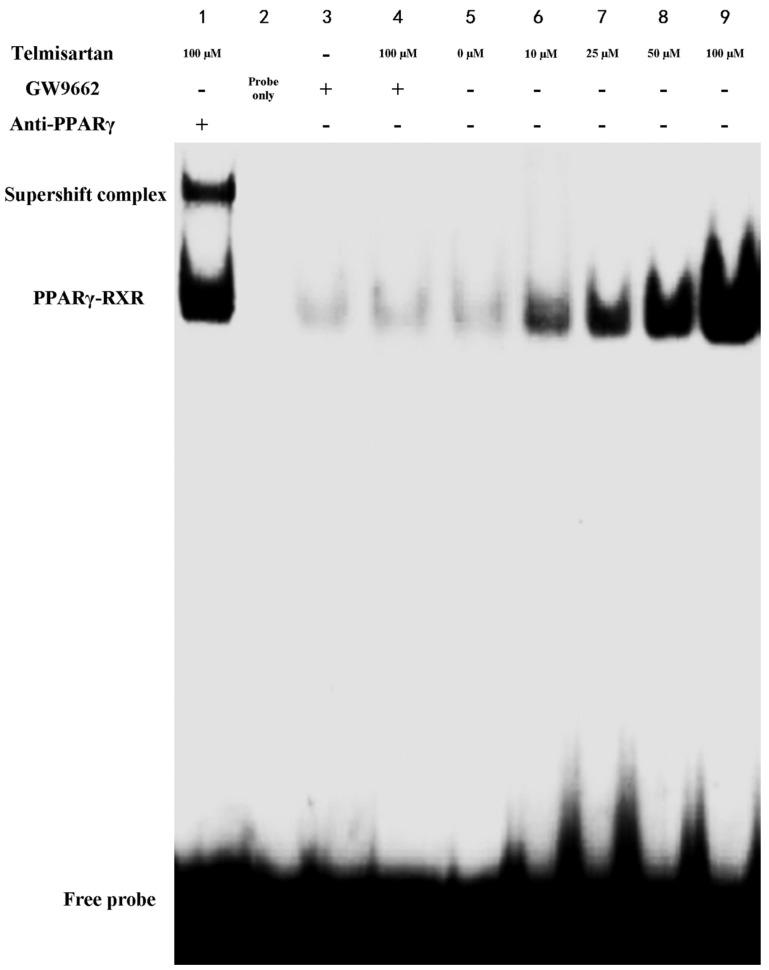
Telmisartan increases DNA binding activity of PPARγ in A549 cells. DNA binding activity of PPARγ in A549 cells treated with 4 concentrations of telmisartan (10, 25, 50 and 100 μM) or GW9662 (10 μM)for 48 h were examined by EMSA. (**1**) Nuclear extracts from 100 μM telmisartan + oligonucleotide probe + anti-PPARγ antibody; (**2**) oligonucleotide probe only; (**3**–**9**) Nuclear extracts from corresponding group + oligonucleotide probe.

EMSA was included to identify PPARγ DNA binding activity in cells. Figure 7 showed that DNA binding activity of PPARγ was dose-dependently increased by telmisartan. Our data also showed that GW9662 blunted the effect of telmisartan on PPARγ DNA binding activity. Otherwise, incubation of the nuclear extracts from 100 μM telmisartan with monoclonal antibody against PPARγ supershifted the complex to the higher molecular weight position (Lane 1 of Figure 7). This result revealed that the shifted complex contains PPARγ. 

### 2.5. Discussion

In the present study, our results indicated that telmisartan dose-dependently inhibits the cell proliferation and the expression of ICAM-1 and MMP-9 through the up-regulation of PPARγ in human non-small cell lung cancer A549 cells. 

Lung cancer has been become the most common cancer globally and the leading cause of cancer deaths in humans. Non-small cell lung cancer (NSCLC) constitutes more than 80% of primary bronchial lung cancers [29,37]. Despite great progress in surgery, chemotherapy, and radiotherapy, the overall survival rate of patients with NSCLC is still not optimistic [37].

Telmisartan, an angiotensin II type 1 receptor blocker (ARB), is usually used for the treatment of hypertension, coronary artery disease (CAD) and diabetic nephropathy [33,34,35]. Recently, studies confirmed that telmisartan and another ARB, irbesartan, act as a ligand for PPARγ [35,36]. Peroxisome proliferator-activated receptors (PPARs) belong to the nuclear hormone receptor family. PPARs consist of three different subtypes, PPARα (NR1C1), PPARβ/δ (NR1C2), and PPARγ (NR1C3) [1,2,3,4,5,6,7,8,9,10]. PPARγ functions as a transcription factor after it heterodimerizes with the retinoid X receptor (RXR) and binds to the specific response elements, which are termed peroxisome proliferating response elements (PPRE) [1,2,3,4,5,6,7]. These PPREs are found in various genes which are involved in lipid metabolism and energy homeostasis, inflammatory regulation, cell proliferation, apoptosis, tumor invasion and metastasis [1,2,3,4,5,6,7]. A wealth of studies have showed that PPARγ was expressed in a variety of tumor cells, and the activation of PPARγ by ligands resulted in either suppression of cell proliferation or induction of apoptosis both *in vivo* and *in vitro* [8,9,10,11,12]. According to our data, we found that the survival rates and cell viabilities of A549 cells were concentration- and time-dependently inhibited by telmisartan (Figure 1 and Figure 2). Additionally, our findings also revealed that the cytotoxic and anti-proliferative properties of telmisartan were blunted by the PPARγ antagonist GW9662 (Figure 1 and Figure 2). These results indicated that the anti-tumor effect of telmisartan was very likely resulted from PPARγ activation in A549 cells. 

Invasion is the one of most critical and fatal characters of malignant tumors. Adhesion of tumor cells to endothelial cells is an essential and initial step of tumor invasion and metastasis [13,14,15,16,17]. Intracellular adhesion molecule-1 (ICAM-1), belonging to the immunoglobulin superfamily, is an inducible 80- to 110-kDa transmembrane glycoprotein [18,19,20]. ICAM-1 is traditionally known to be critical for mediating the interaction of leucocytes with endothelial cells in inflammation [18,19,20]. Meanwhile, some studies also found that many adhesive molecules, including ICAM-1 and vascular adhesion molecule-1 (VCAM-1), can be considered to be crucial for the endothelial adhesion of tumor cells [18,19,20]. Yu *et al.* reported that lung cancer (A549) cell invasion could be suppressed by antibody blockade of ICAM-1 or by inhibition the expression of ICAM-1 [21]. Another study also found that ICAM-1 was essential for lung cancer invasion and indicated that ICAM-1 was a biologically based therapy target for lung cancer [22]. Furthermore, it was reported that overexpression of ICAM-1 was associated with a greater risk of advanced lung cancers (stages III and IV) and more aggressive behaviors in other epithelial-derived tumors including melanoma, gastric cancer and breast cancer [21]. Besides, the study also reported that soluble ICAM-1 (sICAM-1) levels had prognostic value for survival and predictive significance for response during chemotherapy in patients with NSCLC [23]. On the other hand, a number of researches confirmed that PPARγ contributed substantially to the regulation of ICAM-1 under a variety of conditions [24,25,26]. In the current study, our data found that the mRNA and protein expression of ICAM-1 was suppressed by telmisartan in a dose dependent manner. Additionally, the suppressive effect of telmisartan on the expression of ICAM-1 was abrogated by GW9662. These data suggested that ICAM-1 was inhibited by telmisartan, possibly through PPARγ activation in A549 cells. 

Intrapulmonary and distant metastases are the principal cause of death from lung cancer. Degradation of the extracellular matrix (ECM), the key process of metastasis, makes tumor cells invade local organs and tissues, intravasate and extravasate blood vessels, promoting metastasis to secondary tumor sites [27,28,29]. This process is primarily induced by the activity of proteinases secreted by the tumor cells. Matrix metalloproteinases (MMPs), a large family of calcium-dependent zinc-containing endopeptidases, are responsible for the degradation of the ECM, including gelatin, collagens, proteoglycan, matrix glycoproteins, and elastins, in the processes of tumor metastasis [27,28,29,30,31]. Research has demonstrated that higher expression of MMPs is often associated with a poor prognosis for the patients with malignant tumors, such as breast cancer, gastric cancer, lung cancer and hepatic cancer [27,28]. Meanwhile, according to the data, MMPs have been considered as a promising target for cancer therapy and many synthetic and natural MMP inhibitors (MMPIs) have been found or developed, and have been tested in clinical trials [27]. Among MMPs, MMP-9 plays critical role in tumor cell invasion and metastasis by degradation type IV collagen, one of major components of the ECM [27,28,29]. Tang *et al.* found that lung cancer cell invasion and metastasis were enhanced by the up-regulation of MMP-9 in A549 and H1299 cells [38]. Otherwise, some studies also confirmed that the cell proliferation, growth, invasion and metastasis were notably suppressed through inhibition or down-regulation of MMP-9 in lung cancer and other solid cancers [28,29,30]. Simultaneously, Liu *et al.* revealed that PPARγ activation was essential for the regulation of MMP-9 in human myeloid leukemia cells [32]. In our study, we found that the mRNA and protein of MMP-9 was dose-dependently inhibited by telmisartan in A549 cells. Furthermore, GW9662 blunted the inhibitive effect of telmisartan on the expression of MMP-9. These data indicated that MMP-9 was inhibited by telmisartan probably via PPARγ activation in A549 cells. Reasonably, the mRNA and protein expression of PPARγ was evaluated in our study. Our data indicated that PPARγ expression was up-regulated by telmisartan in a dose dependent manner in A549 cells. Otherwise, the EMSA result also showed that DNA binding activity of PPARγ was dose-dependently increased by telmisartan in A549 cells. Unsurprisingly, our data also revealed that telmisartan induced PPARγ expression and DNA binding activity in A549 cells were all abrogated by the PPARγ antagonist GW9662. These data indicated that telmisartan was an effective PPARγ modulator, which could up-regulate the expression and DNA binding activity of PPARγ, in lung cancer A549 cells. 

Therefore, these data indicated that the anti-tumor property of telmisartan possibly results from activation of PPARγ in A549 cells. However, further studies should be carried out to verify the anti-tumor value of telmisartan *in vivo* and in clinic experiments. Meanwhile, the involved signal pathways of telmisartan in PPARγ activation should be elucidated in the future as well. 

## 3. Experimental

### 3.1. Reagents

A549 cells were purchased from American Type Culture Collection (ATCC, Manassas, VA, USA). Telmisartan was obtained from Sigma Chemical Co (St. Louis, MO, USA). GW9662 (a PPARγ antagonist) was purchased from Cayman Chemicals (St Louis, MO, USA). The MTT assay kit was purchased from Promega (Madison, WI, USA). Trizol reagent was obtained from Invitrogen (Grand Island, NY, USA). Anti-β-actin (mouse anti-human IgG), anti-PPARγ (mouse anti-human IgG), anti-ICAM-1 (mouse anti-human IgG), and anti-MMP-9 (mouse anti-human IgG) antibodies were all purchased from Santa Cruz Biotechnology, Inc (Santa Cruz, CA, USA). The Lightshift kit from Pierce (Rockford, IL, USA) was used for EMSA. PPAR consensus oligonucleotides for EMSA were also obtained from Santa Cruz Biotechnology, Inc. 

### 3.2. Cell Culture, MTT Assay and Trypan Blue Exclusion

A549 cells were cultured in RPMI 1640 supplemented with penicillin (100 IU/mL), streptomycin (100 mg/mL), and 10% heat-inactivated fetal bovine serum in a humidified atmosphere of 95% air and 5% CO_2_ at 37 °C. A549 cells were then plated at a density of 5 × 10^3^ cells per well in a 96-well plate. Cells were treated with four different concentrations of telmisartan (10, 25, 50 and 100 μM) or GW9662 (10 μM) in 0.5% DMSO. Forty-eight hours after interventions, protein and mRNA were extracted from cells. The cytotoxic and anti-proliferative effects of telmisartan were determined by MTT assay and trypan blue exclusion assay, at 4 specified time points (0, 24, 48 and 72 h). For MTT assay, survival rate (%) = (OD sample/OD control) × 100 (%) [37]. For the trypan blue exclusion assay, cell viability (%) = (unstained cells of sample/unstained cells of control) × 100 (%) [39].

### 3.3. RT-PCR (Reverse Transcription-Polymerase Chain Reaction)

Total RNA was isolated from the A549 cells with Trizol reagent. PCR was performed with a DNA thermal cycler in a 50-μL reaction volume, containing 5 μL 10 × Taq Buffer, 4 μL of 2.5 mM dNTP, 4 μL of 25 mM MgCl_2_, 2 μL each forward and backward primers, 0.5 μL Taq polymerase, and 2 μL cDNA template, for 35 cycles by using a GeneAmp PCR system 9700 (Applied Biosystems, Foster City, CA, USA). The primer sequences were as follows: ICAM-1, (forward) 5'-CCTCACACTTCACTGTCACCT-3' and (reverse) 5'-CGTGCCGCACTGAACTGGAC-3'; MMP-9, (forward) 5'-ATGAGTTCGGCCACGCGCTGGGCTT-3' and (reverse) 5'-TGCCGGTGATGACACGGAAACTCAG-3'; PPARγ, (forward) 5'-CTATGGAGTTCATGCTTGTG-3' and (reverse) 5'-GTACTGACATTTATTT-3'; β-actin, (forward) 5'-CTCCATCCTGGCCTCGCTGT-3' and (reverse) 5'-GCTGTCACCTTCACCGTTCC-3'. The β-actin housekeeping gene was used as an internal control.

### 3.4. Western Blotting

Western blotting was used to measure the protein content. A549 cells were washed twice with cold PBS. Cells were then lysed with SDS sample buffer containing 50 mM Tris (pH 7.4), 2% SDS (wt/vol), 5% 2-mercaptoethanol, and 10% glycerol. Cell homogenates were centrifuged at 10,000 rpm at 4 °C for 60 min. The protein concentrations for each sample were measured with a bicinchoninic acid assay kit by using BSA as the standard (Pierce). Protein lysates (150 μg) from each sample were subjected to SDS-PAGE on a 10% acrylamide gel, and the separated proteins were transferred onto a PVDF membrane. After incubation for 1 h in blocking solution (5% dry milk in Tris-buffered saline with Tween 20) at room temperature, the membrane was incubated for 24 h with anti-β-actin (1:1,000), anti-ICAM-1 (1:700), anti-MMP-9 (1:800), or anti-PPARγ (1:600) at 4 °C. The secondary antibody (horseradish peroxidase-conjugated goat anti-mouse immunoglobulin) was added at a 1:10,000 dilution and incubated at room temperature for 1 h. Peroxidase labeling was detected with the enhanced chemoluminescence western blotting detection system (Amersham Pharmacia Biotech, Piscataway, NJ, USA) and analyzed by densitometry. The relative protein level was normalized to β-actin. 

### 3.5. Electrophoretic Mobility Shift Assay (EMSA)

According to the previous study, EMSA was performed [37]. In brief, nuclear extracts were isolated from A549 cells with Nuclear Extract Kit (Activemotif, Carlsbad, CA, USA). The PPAR consensus oligonucleotides (5'-CAAAACTAGG TCAAAGGTCA-3'/5'-GTTTTGATCCAG TTTCCAGT-3') was used. Binding reactions were performed using a LightShift Chemoluminescent EMSA Kit (Pierce). The reaction mixtures (10 μL) containing about 5 μg nuclear extracts and 2 pmol of oligonucleotide probe were incubated for 20 min at RT. Specific binding was confirmed by a 200-fold excess of unlabeled probe used as a specific competitor. Protein DNA complexes were separated on a 6% non-denaturing acrylamide gel, transferred to positively charged nylon membranes, and cross-linked using a Stratagene crosslinker (Stratagene, La Jolla, CA, USA). Band shift was visualized with a streptavidin-horseradish peroxidase through chemoluminescent detection. 

### 3.6. Statistical Analysis

Statistical analyses were performed with SPSS version 17.0 software. All data in our study were showed as mean ± SD. A Student’s t-test was used to measure significance, where *p* < 0.05 was considered to be statistically significant. 

## 4. Conclusions

Taken together, our findings confirmed that telmisartan inhibited the expression of ICAM-1 and MMP-9 at the transcriptional level in A549 cells, very likely through the up-regulation of PPARγ synthesis. 
